# The cost of care for children hospitalised with Invasive Group A Streptococcal Disease in Australia

**DOI:** 10.1186/s12913-021-07265-8

**Published:** 2021-12-14

**Authors:** Natasha K. Brusco, Jane Oliver, Alissa McMinn, Andrew Steer, Nigel Crawford

**Affiliations:** 1 Alpha Crucis Group, Health Economics, Langwarrin, Australia; 2 Rehabilitation, Ageing and Independent Living (RAIL) Research Centre, School of Primary and Allied Health Care, Monash University, Peninsula Campus, Frankston, Australia; 3 College of Science Health and Engineering, La Trobe University, Bundoora, Victoria Australia; 4 Tropical Diseases Research Group, Murdoch Children’s Research Institute, Melbourne, Australia; 5 Peter Doherty Institute for Infection and Immunity, University of Melbourne, Melbourne, Australia; 6 Murdoch Children’s Research Institute, Royal Children’s Hospital, Melbourne, Australia; 7 Department of Paediatrics, University of Melbourne, Melbourne, Victoria Australia; 8 Murdoch Children’s Research Institute and Department of General Medicine, Royal Children’s Hospital, Melbourne, Australia

**Keywords:** Economic evaluation, Infectious disease, Invasive group a streptococcal disease, Cost burden, Surveillance

## Abstract

**Background:**

Invasive Group A Streptococcal (iGAS) disease exerts an important burden among Australian children. No Australian hospitalisation cost estimates for treating children with iGAS disease exist, so the financial impact of this condition is unknown.

**Aim:**

To determine the minimum annual healthcare cost for children (< 18 years) hospitalised with iGAS disease in Australia from a healthcare sector perspective.

**Methods:**

A cost analysis including children with laboratory-confirmed iGAS disease hospitalised at the Royal Children’s Hospital (Victoria, Australia; July 2016 to June 2019) was performed. Results were extrapolated against the national minimum iGAS disease incidence. This analysis included healthcare cost from the 7 days prior to the index admission via General Practitioner (GP) and Emergency Department (ED) consultations; the index admission itself; and the 6 months post index admission via rehabilitation admissions, acute re-admissions and outpatient consultations. Additional extrapolations of national cost data by age group, Aboriginal and Torres Strait Islander ethnicity and jurisdiction were performed.

**Results:**

Of the 65 included children, 35% (*n* = 23) were female, 5% (*n* = 3) were Aboriginal and Torres Strait Islander, and the average age was 4.4 years (SD 4.6; 65% aged 0–4). The iGAS disease related healthcare cost per child was $67,799 (SD $92,410). These costs were distributed across the 7 days prior to the index admission via GP and ED consultations (0.2 and 1.1% of total costs, respectively), the index admission itself (88.7% of the total costs); and the 6 months post index admission via rehabilitation admissions, acute re-admissions and outpatient consultations (5.3, 4.5 and 0.1% of total costs, respectively). Based on a national minimum paediatric incidence estimation of 1.63 per 100,000 children aged < 18 (95%CI: 1.11–2.32), the total annual healthcare cost for children with iGAS in 2019 was $6,200,862. The financial burden reflects the overrepresentation of Aboriginal and Torres Strait Islander people in the occurrence of iGAS disease. Costs were concentrated among children aged 0–4 years (62%).

**Conclusion:**

As these cost estimations were based on a minimum incidence, true costs may be higher. Strengthening of surveillance and control of iGAS disease, including a mandate for national notification of iGAS disease, is warranted.

**Trial registration:**

The current study is a part of ongoing iGAS surveillance work across seven paediatric health services in Australia. As this is not a clinical trial, it has not undergone trial registration.

**Supplementary Information:**

The online version contains supplementary material available at 10.1186/s12913-021-07265-8.

## Background

Group A *Streptococcus* (GAS) can produce a range of infections in humans, that vary from superficial to life threatening. Invasive Group A *Streptococcal* (iGAS) disease is a serious condition that occurs when GAS infects a normally sterile body site. iGAS disease has a high burden of long-term morbidity and mortality, including among children [1, 2]. To date there is no licenced vaccine to protect against iGAS disease [3]. While there is a plethora of literature discussing clinical outcomes of iGAS disease, there is a paucity of literature describing the financial costs of iGAS disease at an individual or population level.

The clinical burden of iGAS disease among children in Victoria, Australia, has been well described [1, 2]. However, there is limited knowledge of the financial burden of iGAS disease, in part due to a lack of routinely collected iGAS surveillance data [1, 4]. In the United States, the annual cost associated with all GAS pharyngitis disease in children was estimated between $284 and $684 million 2019 USD (CPI https://www.bls.gov/data/inflation_calculator.htm; $224 and $539 million; 2006 USD), with just over half this sum as direct healthcare costs and the remainder as indirect costs, including lost productivity through parental time off work [5]. In New Zealand, the annual direct healthcare costs associated with all GAS disease across children and adults was estimated at $46 million 2019 USD (CPI https://www.bls.gov/data/inflation_calculator.htm and purchasing power parities (PPP) https://data.oecd.org/conversion/purchasing-power-parities-ppp.htm#indicator-chart; $30 million; 2015 NZD) [6].

GAS infection sequalae include serious conditions such as acute rheumatic fever and rheumatic heart disease, acute post-streptococcus glomerulonephritis [4] and toxic shock syndrome [2]. Internationally, the prevalence of GAS infections has demonstrated within-country differences with a greater burden on migrant and Indigenous-minority populations. This is particularly true in Australia where there are very high rates of rheumatic fever, rheumatic heart disease and glomerulonephritis among remote Aboriginal and Torres Strait Islander peoples [4].

The lack of routine surveillance for iGAS disease limits appropriate development of health policy. It is essential that health services have access to robust and recent cost data to inform future healthcare planning. While there is no clear picture of the current cost of iGAS disease for children in Australia, epidemiological analyses [1, 2] indicate that health-related costs are likely to be significant. In absence of a national surveillance system, the minimum national Australian incidence rate of iGAS disease among children aged less than 18 years was estimated as 1.63 per 100,000 (95%CI: 1.11–2.32) between June 2016 to July 2018 [1]. Calculation of this rate used data collected through the Paediatric Active Enhanced Disease Surveillance (PAEDS) Network, a voluntary case notification system which included reporting from seven major Australian children’s hospitals.

To contribute to filling this evidence gap, we designed a study to determine the average healthcare costs, from a healthcare sector perspective, for children hospitalised with iGAS at the Royal Children’s Hospital (RCH). We used these data, in combination with the minimum iGAS incidence for Australia, to estimate annual healthcare costs which are likely to be below the true value as a consequence of minimal estimates of incidences. This included healthcare cost from the 7 days prior to the index admission via General Practitioner (GP) and Emergency Department (ED) consultations; the index admission itself; and the 6 months post discharge from the index admission via rehabilitation admissions, acute re-admissions and outpatient consultations. The aim of this study was to determine the minimum annual healthcare cost for children (< 18 years) hospitalised with iGAS disease in Australia from a healthcare sector perspective.

## Methods

RCH is a major tertiary children’s hospital in metropolitan Melbourne [7]. Melbourne is the state capital of Victoria, Australia, a high-income country in the south Pacific [8]. Victoria is the second most populous Australian state. At the 2016 national census, 5.9 million people lived in Victoria; approximately one-quarter of the national population [9].

We conducted a cost analysis including children aged < 18 years with laboratory-confirmed iGAS disease hospitalised at RCH between 1 July 2016 to 30 June 2019 (including complete admissions only), with results extrapolated against the national minimum paediatric iGAS disease incidence [1]. There was no intervention and no comparator group.

The study was reported according to the consolidated health economic evaluation reporting standards (CHEERS) checklist for economic evaluations [10]. Ethics approval was obtained from the Royal Children’s Hospital Human Research Ethics Committee (HREC/66284/RCHM-2020-221,038) as an additional study under the broader PAEDS platform. Written informed consent was obtained from the parents of the children involved in the PAEDS platform study, to participate in the original surveillance analysis which collected demographic, clinical and 6 months follow up data [1]. The Royal Children’s Hospital Human Research Ethics Committee then provided a waiver of informed consent to complete this post-hoc economic evaluation which used the same surveillance study data in addition to obtaining individual cost data from the hospital administrative system.

Cost data from all eligible children admitted to an acute care ward during the study period were included. Acute care wards included general acute wards, the ED and the Intensive Care Unit (ICU). Where available, clinical and cost data from the 6 months following the index admission discharge date were included, as were data from the 7 days preceding the index admission; in order to represent a healthcare sector perspective. Cost data collected prior to the 2018–19 financial year were inflated by the Consumer Price Index Australia [11] for a net present value of 2018–19 $AUD. An a priori sub-group analysis was conducted for Aboriginal and Torres Strait Islander children as it is known that this group experiences a higher disease burden compared to other Australian children [4].

### Clinical outcomes

Clinical events relating to the iGAS disease index admission included presentation to a GP or ED in the 7 days prior to hospitalisation, as well as the ED triage category, respiratory support provided, surgical procedures conducted, ICU admission within 24 h of presentation, and an ICU admission during the index hospital admission. In addition, discharge destination (home, inpatient rehabilitation, outpatient rehabilitation, hospital in the home utilisation, hospital transfer), and outcome at discharge categorised as disability (i.e. mobility, intellectual disability, amputation, other), deficit (i.e. cognitive, fatigue, physical or other) or death were reported.

Clinical outcomes within 6 months of discharge included hospital re-admissions, outpatient appointments, ongoing health issues, and the patient’s current health status compared to their pre-iGAS disease status [1]. Clinical outcomes have been previously reported in an aggregate analysis that combined results from for this cohort with six other major paediatric centres in Australia, and as a Victoria-specific analysis combining results for the state’s two major paediatric centres [1, 2].

### Resources and costs

The primary outcome was the total healthcare cost per child admitted to RCH with laboratory-confirmed iGAS disease. The secondary outcome was the extrapolation of cost data across the Australian paediatric population using the minimum iGAS incidence for children < 18 years [1]. This cost extrapolation was mapped against a major Australian children’s hospitals’ data from the PAEDS Network to estimate annual healthcare costs which, as previously noted, are likely to be below the true value as a consequence of minimal estimates of incidences.

Resource and cost data were collated prospectively and retrospectively through two pre-existing data sources following identification of eligible patients. The first data source was clinical data which accompanied iGAS case notifications [1, 2]. The second was an extract of individual patient-level cost and utilisation data obtained from the RCH Decision Support Unit for the index admission, as well as any re-admissions, clinical events and outcomes in the 6 months post-discharge. Individual cost categories are presented for the ICU admission, acute ward admission, hospital-in-the-home admission, blood products and utilisation of pathology, imaging, pharmacy, theatre, allied health and other.

### Extrapolation

Extrapolations of cost data were used to estimate the national minimum cost of iGAS disease nationally by: age group, Aboriginal and Torres Strait Islander ethnicity and by jurisdiction. Australian Bureau of Statistics (ABS) general population data were used [12] in addition to ABS data specific to Aboriginal and Torres Strait Islander people [13]. To complete the extrapolation by jurisdiction we assumed that the iGAS incidence for Tasmania was the same as Victoria, and the incidence for Australian Capital Territory (ACT) was the same as New South Wales.

The cost per person was then extrapolated across the Australian population using the national minimum paediatric iGAS incidence from July 2016 to 30 June 2018 inclusive [1]. The overall incidence for this period was applied to the 2019 population (12 month time horizon), as were the sub-group incidence (i.e. age group, ethnicity, jurisdiction). When the sub-group incidence for 2019 were combined to produce an overall incidence, there was an expected small variation from the July 2016 to 30 June 2018 overall incidence. This was because the denominator (population size) for each sub-group varied slightly between 2019 and the July 2016 to 30 June 2018 period, placing more or less weight on particular sub-group incidence, and therefore impacting the combined incidence. This was compounded in the jurisdiction analysis by attributing an incidence to Tasmania and ACT, as both were previously un-represented in incidence estimations.

### Analysis

We report the unit definition, unit cost, unit quantity and total cost for each resource, presented as a mean cost and standard deviation. Individual patient utilisation and cost data from the RCH Decision Support Unit were used to represent hospital utilisation and cost. Self-reported healthcare utilisation survey data were used for resource and cost allocation relating to GP and ED consultations in the 7 days prior to the index admission, and for outpatient consultations in the 6 months following the index admission. Missing data for the outpatient consultations were managed by taking the mean value of available data (*n* = 42, 65%) and imputing it into the missing data fields (*n* = 23, 35%). Each of the units were combined together to present a total cost per child for the 7 days prior the index admission, during the index admission, and for the 6 months post index admission. Extrapolations corresponded to the overall 2019 population as well as sub-groups based on age or jurisdiction. A subgroup analysis specific to Aboriginal and Torres Strait Islander children was completed.

A sensitivity analysis was used to adjust the self-reported healthcare utilisation costs (GP, ED and outpatient consultations) due to the risk of recall bias. Recall bias occurs when person reporting the occasions of healthcare services utilised mis-remembers / reports events that did not occur (over-reporting) or forgets / does not report events that did occur (under-reporting) [14]. It has been reported that over six-months the variation in over and under-reporting can be as high as 35 and 36%, respectively [14]. To account for this recall bias, self-reported data (GP, ED and outpatient consultations) was deflated by 35% and inflated by 36% in a sensitivity analysis. Analyses were conducted using Microsoft Excel 2019 and SPSS Version 25 [15]. Differences between reported findings were considered statistically significant if *p* < 0.05 using an independent t-test.

## Results

### The minimum annual healthcare cost for children hospitalised with iGAS disease at RCH

Sixty-five patients with iGAS disease were admitted to RCH and included in the analyses. Thirty-five percent were female, 5% identified as Aboriginal and Torres Strait Islanders, and the mean age was 4.44 years (SD 4.60) with 65% aged ≥4 years (Table 1).

**Table 1:** Demographic and clinical characteristics of children < 18 years admitted for iGAS (July-2016 to June-2018)

Children	Number (proportion)
Total	65 (100.0%)
Female	23 (35.4%)
Male	42 (64.6%)
Aboriginal and Torres Strait Islander	3 (4.6%)
Age group	
< 1 year	17 (26.2%)
1–4 years	25 (38.5%)
5–9 years	16 (24.6%)
10–14 years	3 (4.6%)
15–17 years	4 (6.2%)
**Seven days prior to index admission (*n*** **= 65)**	
Prior ED consultations at another hospital	45 (69.2%), range 0 to 5, mean = 0.98 (SD 0.94), total = 64
Prior GP consultations	42 (64.6%),range = 0 to 5, mean = 1.03 (SD 1.13), total = 67
**During index admission (*n*** **= 65)**	
ED presentation – index hospital	55 (84.6%)
*ED triage Category 1*	*1 (1.5%)*
*ED triage Category 2*	*13 (20.0%)*
Respiratory support provided	28 (43.1%)
Surgical procedures conducted	44 (67.7%)
*ICU admission within 24 h of presentation*	*20 (30.8%)*
ICU admission during the index hospital admission; days admitted to ICU	27 (41.5%), LOS range 0 to 29, mean 3.72 (SD 7.50), total = 242
IVIG Administered	13 (20.0%)
Discharge destination	
home	38 (58.5%)
inpatient rehabilitation	5 (7.7%)
hospital in the home	15 (23.1%)
transfer to another hospital	6 (9.2%)
Disability on discharge	
deceased	1 (1.5%)
deficit (can improve)	21 (32.3%)
disability (permanent)	1 (1.5%)
deficit and disability	2 (3.1%)
full recovery	38 (58.5%)
Data not available	2 (3.1%)
Length of stay index admission, days, mean (SD), range	14.29 (SD 11.86), range = 1 to 63
**6 months post index admission**	
Ongoing health issues	
Yes, ongoing issues	8 (12.3%)
No, full recovery	34 (52.3%)
Data not available	23 (35.4%)
Current health status	
Normal	27 (41.5%)
Mild	11 (16.9%)
Moderate	2 (3.1%)
Severe	1 (1.5%)
Data not available	24 (36.9%)
Rehabilitation length of stay, days, mean (SD), range	1.74 (SD6.47), range = 0 to 30
Re-admissions length of stay, days, mean (SD), range	1.42 (SD 4.91), range = 0 to 25

*ED* emergency department, *GP* general practitioner doctor, *ICU* intensive care unit, *IVIG* intravenous immunoglobulin, *LOS* length of stay

### Clinical outcomes

In the 7 days prior to the index admission, 42/65 patients presented to a to an ED (65% of patients; 64 presentations in total) and 45/65 patients presented to a GP (69% of patients; 67 presentations in total). During the index admission, 27 patients (42%) required care in an ICU and 44 (68%) had a surgical procedure, and one child died. Following a mean length of stay in the acute ward of 14 days (SD: 12 days), 5 patients (8%) required inpatient rehabilitation, and 24 (37%) had a deficit and / or disability. At 6 months after discharge, 8 patients (12%) had ongoing health issues related to iGAS. Twelve patients (18%) were re-admitted to RCH with a length of stay from 1 to 25 days (Table 1).

### Resources and costs

The estimated average iGAS disease-related healthcare cost per child was $67,799 (SD $92,410). These costs were distributed across the 7 days prior to the index admission via GP and ED consultations (0.2 and 1.1% of total costs, respectively), the index admission itself (88.7% of the total costs); and the 6 months post index admission via rehabilitation admissions, acute re-admissions and outpatient consultations (5.3, 4.5 and 0.1% of total costs, respectively). The combined cost of healthcare utilisation for the 65 patients was $4,406,931 (Table 2). Given the higher prevalence amongst males, a comparison of costs was completed between males and females. The mean total cost of healthcare for iGAS disease was $57,217 (SD $80,632) for males (*n* = 42) compared to $87,123 (SD $110,098), for females (*n* = 23); mean difference $-29,906 (95%CI: -$77,596 to $17,784; *p* = 0.215).

**Table 2:** Resource unit definition, unit cost, unit quantity and total cost for each resource

	Unit definition and data source	Unit cost (if available, fixed value per resource utilised); number of patients	Mean cost per patient (***n*** = 65)	Total cost across all patients (***n*** = 65)
**Prior to index admission (*n*** **= 65); unit cost is per consultation**				
Prior ED consultations	1x ED consultation in the 7 days prior to the index admission. This includes an ED admission with a direct transfer to the index hospital. Source: Independent Hospital Pricing Authority, Public Sector, Round 22 (Financial Year 2017–18) multiplied by CPI for 2018/19 costs [16]	$716.62; *n* = 64 (range = 0 to 5 per patient)	$705.60 (SD $676.20)	$45,863.48
Prior GP consultations	1x GP appointment at Level D Item 413 Source: Based on MBS Fees 201905-MBS.pdf (mbsonline.gov.au) for the 2018/19 fee schedule, p255	$123.55; *n* = 67 (range = 0 to 5 per patient)	$127.35 (SD $139.80)	$8277.85
**During index admission (*n*** **= 65); unit cost is the average cost per child (Source: From RCH administrative dataset)**				
TOTAL INDEX ADMISSION	All costs associated with the index admission	Variable; n = 65	$60,112.13 (SD $79,072.41)	$ 3,907,288.38
ICU admission	ICU costs associated with the index admission	Variable; *n* = 27	$23,155.92 (SD $47,258.65)	$ 1,505,134.87
Acute ward admission	Acute ward costs associated with the index admission (nursing and medical costs and supplies)	Variable; n = 64	$16,314.36 (SD $15,695.65)	$ 1,060,433.46
HITH admission	HITH costs associated with the index admission	Variable; *n* = 17	$1154.63 (SD $2890.21)	$ 75,050.78
Blood products	Blood product costs associated with the index admission (including IVIG)	Variable; n = 20	$1065.59 (SD $2826.56)	$ 69,263.04
Pathology utilisation	Pathology costs associated with the index admission	Variable; *n* = 60	$1851.36 (SD $2308.96)	$ 120,338.63
Imaging utilisation	Imaging costs associated with the index admission	Variable; *n* = 54	$3508.25 (SD $6187.47)	$ 228,035.98
Pharmacy utilisation	Pharmacy costs associated with the index admission	Variable; n = 65	$1523.60 (SD $2283.74)	$ 99,033.83
Theatre utilisation	Theatre and surgery costs associated with the index admission	Variable; *n* = 49	$4835.93 (SD $5472.89)	$ 314,335.20
Allied Health utilisation	Allied Health costs associated with the index admission	Variable; n = 64	$1289.94 (SD $2332.85)	$ 83,846.00
Other	Any other index admission costs not previously specified.	Variable; n = 64	$5412.56 (SD $5269.33)	$ 351,816.57
**Inpatient re-admissions (6 months post index admission) (*n*** **= 65); unit cost is the average cost per child**				
Rehabilitation inpatient admission directly after the index admission	Per diem rate for all admission costs associated with re- admissions 0-6 months post index admission Source: Modelled data from the RCH administrative dataset	Variable, n = 5	$3625.89 (SD $13,401.38)	$235,682.70
Re-admission to the index hospital	Per diem rate for all admission costs associated with re- admissions 0-6 months post index admission Source: Modelled data from the RCH administrative dataset	Variable, *n* = 12	$3030.84 (SD $11,187.71)	$197,004.87
**Outpatient services (6 months post index admission) (*n*** **= 65); unit cost is per consultation**				
Outpatient consultations	1x outpatient consultation for an allied health service or medical review Source: Based on Allied Health MBS Fees 201905-MBS.pdf (mbsonline.gov.au) for the 2018/19 fee schedule, p1288	$62.25; *n* = 206 (range = 0 to 45 per patient)	$197.13 (SD 409.37)	$12,813.60
**COMBINED HEALTHCARE COSTS HEALTHCARE COSTS PRE-ADMISSSION, INDEX ADMISSION AND THE 6 MONTHS FOLLOWING ADMISSION (*n*** **= 65)**				
Total healthcare costs	All reported healthcare costs in the 7 days prior to the index admission, during the index admission, and in the 0-6 months post index admission	Variable; n = 65	Mean $67,798.94 (SD$92,409.74) Range $2831.45 to $492,347.38 TOTAL $4,406,931.08	

*ED* emergency department, *GP* general practitioner doctor, *HITH* hospital in the home, *ICU* intensive care unit, *IVIG* intravenous immunoglobulin, *LOS* length of stay

A sub-group analysis was completed for the 3 Aboriginal and Torres Strait Islander patients. On a per-patient basis, comparing Aboriginal and Torres Strait Islander and other Australians children, the mean total cost of healthcare for iGAS disease was similar with $68,444 (SD $52,811) compared to $67,768 (SD $94,170), respectively; mean difference $676.69 (*p* = 0.985). While overall mean costs were similar, Aboriginal and Torres Strait Islander patients had significantly higher costs associated with prior ED consultations (mean difference: $1013.29; 95%CI: $249.64 to $1776.93; *p* = 0.039) and significantly lower costs associated with rehabilitation admissions immediately post index admission (mean difference: -$3801.33; 95%CI: -$7281.06 to -$321.61; *p* = 0.033, Additional Table 2). The 3 Aboriginal and Torres Strait Islander patients were also compared via case match to other Australians children where they were matched on age, gender and underlying co-morbidities. The mean total cost of healthcare for iGAS disease was $68,444 (SD $52,811) for the Aboriginal and Torres Strait Islander patients (*n* = 3) compared to $102,967 (SD $131,386), for the matched other Australians children (n = 3); mean difference $34,523 (95%CI: $-192,463 to $261,509; *p* = 0.695).

To account for recall bias of the self-reported healthcare utilisation (GP, ED and outpatient consultations), the self-reported healthcare utilisation was inflated by 36% and deflated by 35%, the per-patient cost of these 3 combined resources (valued at $1030, SD $862) increased to $1401 (SD $1172) and decreased to $763 (SD $639), respectively. Similarly, combined with all healthcare utilisation for the 7 days prior to the index admission, during the index admission, and for the 6 months after discharge, the per patient- cost (valued at $67,799, SD $92,410) increased to $68,170 (SD $92,542) and decreased to $67,532 (SD $92,315), respectively.

### The minimum annual national cost of healthcare for children with iGAS disease

**Fig. 1:**
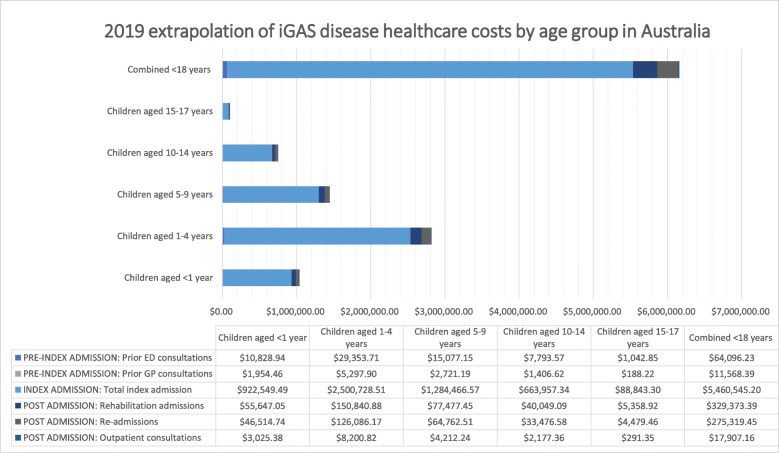
2019 extrapolation of iGAS disease incidence and healthcare costs by age group in Australia

Based on a national minimum paediatric incidence estimation of 1.63 per 100,000 children aged < 18 (95%CI: 1.11–2.32; based on 2016 to 2018 data) [1], the total annual healthcare cost for children with iGAS in 2019 was $6,200,862 (Fig. 1, Additional Table 1), with costs concentrated among children aged 0–4 years (62%). However, the incidence varied slightly when the sub-group incidence (i.e., age group, ethnicity, jurisdiction based on 2016 to 2018 data) were applied to the 2019 sub-groups and re-totalled to produce an overall incidence. This was due to the denominator (population size) for each sub-group varying between 2019 and the 2016 to 2018 period, placing more or less weight on particular sub-group incidence, and therefore impacting the combined incidence.

Based on the age sub-group incidence from 2016 to 2018 being applied to the 2019 age sub-groups, then re-totalled to produce an overall incidence for 2019, an incidence of 1.62 per 100,000 aged < 18 was reported (Fig. 1, Additional Table 1). Based on this incidence, the total national healthcare cost for children with iGAS in 2019 in Australia was $6,158,810, with 86.2% of this total cost concentrated among children aged less than 10 years, and 45.8% concentrated among children aged one to 4 years (Fig. 1, Additional Table 1).

Based on the jurisdiction sub-group incidence from 2016 to 2018 being applied to the 2019 jurisdiction sub-groups, in addition to adding an assumed incidence for ACT and Tasmania (previously unreported), then re-totalled to produce an overall incidence for 2019, an incidence of 1.69 per 100,000 aged < 18 was reported (Additional Table 3). Based on this incidence, the total national healthcare cost for children with iGAS in 2019 in Australia was $6,424,245. The financial burden was highest for Victoria, which contributed 32.8% of the national cost, followed by Queensland, contributing 23.1% (Additional Table 3). Based on the extrapolated incidence of 3.51 per 100,000 for Aboriginal and Torres Strait Islander children, the total national healthcare cost for iGAS disease for this group in 2019 was $820,157 (Additional Table 4) and this represented 13.3% of the total national costs.

## Discussion

A high financial burden was associated with treating paediatric iGAS disease in Australia. This reflects the serious nature of iGAS disease, which often requires prolonged acute hospitalisation, ICU admission and surgery [17], and in this study these elements of care represented 27, 39 and 8% of the total index admission cost, respectively. The disease disproportionately affects younger children, reflected by our finding that 62%, of the financial burden was concentrated among children aged 0–4 years old. While there is a paucity in direct comparison of equivalent spending with other international healthcare systems, the per capita spend on all infectious diseases in Australia ranged from 76% of the per capita cost in the Netherlands to 156% of the per capita costs in Canada [18].

We estimated that the national annual healthcare cost related to iGAS hospital admissions for children in 2019 was $6.2 million in AUD, or $9.1 million 2019 USD PPP. While the Australian cost was limited to the health care sector perspective for children hospitalised with iGAS, comparison is made with the annual cost of all non-hospitalised GAS pharyngitis disease in children ($284 and $684 million 2019 USD), and with the annual direct healthcare costs in New Zealand associated with all GAS disease across children and adults at $46 million 2019 USD [6]. In Australia, the true costs may be considerably higher as this estimation draws on a minimum rate, it is expected that some patients would have needed ongoing follow-up care beyond the 6-month follow-up period, and it is exclusive of indirect costs such as parental loss of income associated with caring for the child which is included in the US study [6]. Based on the US study [6], the inclusion of indirect costs, such as parental lost work, could potentially double the financial burden of the current iGAS in Australia. It is currently unknown how long-term iGAS related disabilities, beyond the 6-month follow up period, would impact the cost estimates, or if long term costs would be covered by the public health system, the National Disability Insurance Scheme, or privately through health insurance or out-of-pocket costs.

This financial burden was concentrated in Victoria and Queensland, which together comprise 56% of the total national burden. There is an opportunity for policymakers in Australia, especially these states, to strengthen policies regarding iGAS disease. iGAS disease is not a nationally notifiable disease, although it is notifiable in Queensland and the Northern Territory. The total annual cost of iGAS index hospitalisation for children in Australia aged less than 4 years was $3.4 million and costs increased to $3.9 million when prior ED and GP costs, re-admissions, rehabilitation admissions and outpatient consultations costs were considered. By comparison the annual national cost of paediatric index hospitalisations with influenza in this age group is $5 million in Australia [19]. Despite a fairly similar financial burden, there is a national mandate to notify all influenza cases to the Federal Government but not for iGAS [20, 21]. The high financial burden observed in these analyses was based on minimum rate estimations and this provides further support for the need for national iGAS patient notification to formally monitor the burden of disease.

We observed that the cost of healthcare for iGAS for Aboriginal and Torres Strait Islander children comprised over 13% of the total national cost, however Aboriginal and Torres Strait Islander children account for only 3% of the total Australian population [9]. Aboriginal and Torres Strait Islander children are over-represented across the spectrum of GAS infections. There is an extremely high burden of GAS impetigo observed in many remote Indigenous Australian communities [22]. Of the approximately 6000 people receiving treatment and monitoring on Australian rheumatic heart disease registers, over 90% are Aboriginal and Torres Strait Islander people [23]. Culturally appropriate interventions to reduce the burden of GAS infections for Aboriginal and Torres Strait Islander children are strongly warranted and our data provide further impetus. There are a range of prevention strategies including prompt treatment of superficial GAS infections before complications arise, provision of contact prophylaxis to household contacts of index iGAS cases [24] and, more broadly, addressing the socially unjust distribution of the determinants of health, including improved access to healthcare and hygienic practices, and reductions in household crowding [25, 26].

The main limitation of our study concerns the small sample size and access to individual patient-level cost data at only one health service (RCH). Although RCH was working in collaboration with 6 other major paediatric centres in Australia who were consistently monitoring and reporting iGAS surveillance data [1], it was not feasible to obtain cost data from these 6 centres. The health service in the current study was one of the top two hospitals contributing to the national iGAS surveillance data [1] and costs were extrapolated to a national level. Our national estimate of annual healthcare costs is likely to be below the true cost burden of iGAS for children in Australia, as a consequence of minimal estimates of incidences. The ethnicity-specific analyses in our study need to be interpreted with care due to the small number of children identified as Aboriginal and Torres Strait Islanders (*n* = 3).

## Conclusion

As these cost estimations of iGAS for children in Australia were based on a minimum incidence, true costs may be higher. Strengthening of surveillance and control of iGAS disease, including a mandate for national notification of iGAS disease, is warranted.

## Supplementary Information

**Additional file 1.**

## Data Availability

The datasets generated and/or analysed during the current study are not publicly available due to a small sample size and the potential for re-identification, but are available from the corresponding author on reasonable request.

## Abbreviations

PAEDS: Paediatric Active Enhanced Disease Surveillance
iGAS: Invasive Group A Streptococcal
GAS: Group A Streptococcus
GP: General Practitioner
ED: Emergency Department
RCH: Royal Children’s Hospital
ICU: Intensive Care Unit
