# Supplementary material for: The cost of care for children hospitalised with Invasive Group A Streptococcal Disease in Australia
Source: BMC Health Serv Res. 2021 Dec 14;21:1340. doi: 10.1186/s12913-021-07265-8 (PMC8670128; doi:10.1186/s12913-021-07265-8)
Supplement: Supplementary file 1 — Additional file 1. [file 12913_2021_7265_MOESM1_ESM.docx]

## Additional files

File format – Microsoft Word

Title (and description) of the data:

- Additional Table 1: 2019 national extrapolation of iGAS disease incidence and healthcare costs by age group in Australia
- Additional Table 2: Cost per child comparing Aboriginal and Torres Strait Islander patients (n=3; 5%) and other Australians (n=62; 95%)
- Additional Table 3: 2019 national extrapolation of iGAS disease incidence and healthcare costs by jurisdiction in Australia
- Additional Table 4: National extrapolation of invasive group A streptococcus disease incidence rates and healthcare costs in 2019 by age group for Aboriginal and Torres Strait Islander children in Australia

Additional Table 1: 2019 extrapolation of iGAS disease incidence and healthcare costs by age group in Australia

|  | **Children aged <1 year** | **Children aged 1-4 years** | **Children aged 5-9 years** | **Children aged 10-14 years** | **Children aged 15-17 years** | **Combined <18 years^1^**  **Rate based on combined 2019 age category incidence** | **Combined <18 years^2^**  **Rate based on historic overall incidence** |
| --- | --- | --- | --- | --- | --- | --- | --- |
| Population (2019) | 302,705 | 1,264,470 | 1,618,776 | 1,555,678 | 869,388 | 5,611,017 | 5,611,017 |
| Incident rate (per 100,000) | 5.07 | 3.29 | 1.32 | 0.71 | 0.17 | 1.62 | 1.63 |
| Impacted population | 15.3 | 41.6 | 21.4 | 11.0 | 1.5 | 90.8 | 91.5 |
| **Prior to index admission** | | | | | | | |
| Prior ED consultations | $10,828.94 | $29,353.71 | $15,077.15 | $7,793.57 | $1,042.85 | $64,096.23 | $64,533.88 |
| Prior GP consultations | $1,954.46 | $5,297.90 | $2,721.19 | $1,406.62 | $188.22 | $11,568.39 | $11,647.38 |
| **During index admission** | | | | | | | |
| Total index admission | $922,549.49 | $2,500,728.51 | $1,284,466.57 | $663,957.34 | $88,843.30 | $5,460,545.20 | $5,497,829.99 |
| **Inpatient re-admissions (6 months post index admission)** | | | | | | | |
| Rehabilitation admissions | $55,647.05 | $150,840.88 | $77,477.45 | $40,049.09 | $5,358.92 | $329,373.39 | $331,622.37 |
| Re-admissions | $46,514.74 | $126,086.17 | $64,762.51 | $33,476.58 | $4,479.46 | $275,319.45 | $277,199.34 |
| **Outpatient services (6 months post index admission)** | | | | | | | |
| Outpatient consultations | $3,025.38 | $8,200.82 | $4,212.24 | $2,177.36 | $291.35 | $17,907.16 | $18,029.43 |
| **TOTAL HEALTHCARE COSTS PRE, INDEX ADMISSION AND THE 6 MONTHS FOLLOWING** | | | | | | | |
| Total healthcare costs | $1,040,520.06 | $2,820,507.97 | $1,448,717.12 | $748,860.57 | $100,204.09 | $6,158,809.82 | $6,200,862.38 |

^1^ Combining the individual age group categories to create an overall population and incident rate for 2019

^2^ Using the 1.63/100,000 overall incidence rate reported by Oliver et al., for the period 1 July 2016 to 30 June 2018 inclusive(1)

Additional Table 2: Cost per child comparing Aboriginal and Torres Strait Islander patients (n=3; 5%) and other Australians (n=62; 95%)

|  | **Aboriginal and Torres Strait Islander patients (n=3), mean cost per patient (SD)** | **Other Australians (n=62), mean cost per patient (SD)** | **Mean difference (Aboriginal and Torres Strait Islander patients minus other Australians), 95% CI** |
| --- | --- | --- | --- |
| **Prior to index admission** | | | |
| Prior ED consultations | $1,672.11 (SD $413.74)  Mean number of consultations 2.33 (SD 0.58) | $658.83 (SD $652.66)  Mean number of consultations 0.92 (SD 0.91) | **$1,013.29 (95%CI: $111.87 to $1,914.70; p=0.039)** |
| Prior GP consultations | $82.37 (SD $71.33)  Mean number of consultations 0.67 (SD 0.58) | $129.53 ($142.24)  Mean number of consultations 1.05 (SD 1.15) | -$47.16 (95%CI: -$194.94 to $100.62; p=0.375) |
| **During index admission** | | | |
| Total index admission | $60,641.24 ($51,236.45);  n=3 (100%) | $60,086.53 (80,460.28);  n=62 (100%) | $554.71 (95%CI: -$111,172.46 to $112,281.89; p=0.987) |
| **Inpatient re-admissions (6 months post index admission)** | | | |
| Rehabilitation inpatient admission directly after the index admission | $0.00 (SD $0.00);  n=0 (0%) | $3,801.33 (SD $13,702.26);  n=5 (8%) | **-$3,801.33 (95%CI: -$7,281.06 to -$321.61; p=0.033)** |
| Re-admission to the index hospital | $5,851.53 (SD $10,135.15);  n=1 (33%) | $2,894.36 (SD $11,293.47);  n=11 (18%) | $2,957.17 (95%CI: -$20,41492 to $26,329.27; p=0.667) |
| **Outpatient services (6 months post index admission)** | | | |
| Outpatient consultations | $197.15 (SD $0.00)  Mean number of consultations 3.17 (SD 0.00) | $197.13 (SD $419.32)  Mean number of consultations 3.17 (SD 6.74) | $0.01 (95%CI: -$106.47 to $106.50; p=1.000) |
| **MEAN VALUE OF COMBINED HEALTH CARE COSTS PRE-ADMISSSION, INDEX ADMISSION AND THE 6 MONTHS FOLLOWING ADMISSION** | | | |
| Total health care costs | Mean $68,444.40 (52,810.79);  Median $81,184.65 (range %10,428.91 to $113,719.65)  n=3 (100%) | Mean $67,767.71 (SD $94,170.46);  Median $40,695.20 (range $2,831.45 to $492,347.38)  n=62 (100%) | MD $676.69 (95%CI: -$111,493.04 to $112,846.42; p=0.985) |

Bold typeface indicates a result is statistically significant (p<0.05); ED = emergency department; GP = general practitioner doctor

Additional Table 3: 2019 national extrapolation of iGAS disease incidence and healthcare costs by jurisdiction in Australia

|  | **ACT** | **NSW** | **NT** | **QLD** | **SA** | **TAS** | **VIC** | **WA** | **Combined^1,2^**  **Rate based on combined 2019 location category incidence** | **Combined^3^**  **Rate based on historic overall incidence** |
| --- | --- | --- | --- | --- | --- | --- | --- | --- | --- | --- |
| Population (<18y 2019) | 94,933 | 1,773,189 | 61,970 | 1,176,850 | 368,545 | 112,530 | 1,419,228 | 602,844 | 5,610,089 | 5,611,017 |
| Incident rate (per 100,000) | 0.65 | 0.65 | 3.99 | 1.86 | 2.99 | 2.19 | 2.19 | 2.27 | 1.69 | 1.63 |
| Impacted population | 0.6 | 11.5 | 2.5 | 21.9 | 11.0 | 2.5 | 31.1 | 13.7 | 94.8 | 91.5 |
| **Prior to index admission** | | | | | | | | | | |
| Prior ED consultations | $435.40 | $8,132.55 | $1,744.67 | $15,445.17 | $7,775.36 | $1,738.89 | $21,930.82 | $9,655.82 | $66,858.68 | $64,533.88 |
| Prior GP consultations | $78.58 | $1,467.80 | $314.89 | $2,787.62 | $1,403.33 | $313.84 | $3,958.18 | $1,742.73 | $12,066.97 | $11,647.38 |
| **During index admission** | | | | | | | | | | |
| Total index admission | $37,093.06 | $692,836.09 | $148,633.43 | $1,315,819.06 | $662,405.35 | $148,140.75 | $1,868,350.71 | $822,607.98 | $5,695,886.44 | $5,497,829.99 |
| **Inpatient re-admissions (6 months post index admission)** | | | | | | | | | | |
| Rehabilitation admissions | $2,237.41 | $41,791.02 | $8,965.39 | $79,368.59 | $39,955.48 | $8,935.67 | $112,696.63 | $49,618.70 | $343,568.89 | $331,622.37 |
| Re-admissions | $1,870.22 | $34,932.64 | $7,494.06 | $66,343.30 | $33,398.33 | $7,469.22 | $94,201.82 | $41,475.71 | $287,185.31 | $277,199.34 |
| **Outpatient services (6 months post index admission)** | | | | | | | | | | |
| Outpatient consultations | $121.64 | $2,272.07 | $487.42 | $4,315.06 | $2,172.27 | $485.81 | $6,127.02 | $2,697.64 | $18,678.93 | $18,029.43 |
| **TOTAL HEALTHCARE COSTS PRE, INDEX ADMISSION AND THE 6 MONTHS FOLLOWING** | | | | | | | | | | |
| Total healthcare costs | $41,836.32 | $781,432.18 | $167,639.86 | $1,484,078.80 | $747,110.11 | $167,084.18 | $2,107,265.17 | $927,798.58 | $6,424,245.20 | $6,200,862.38 |

ACT = Australian Capital Territory; NSW = New South Wales; NT = Northern Territory; QLD = Queensland; SA = South Australia; TAS = Tasmania; VIC = Victoria; WA = Western Australia

^1^ Combining the individual state/territory categories to create an overall population and incident rate

^2^ Small difference in the overall population size is noted (5,610,089 compared to 5,611,017) due to the use of different ABS datasets

^3^ Using the 1.63/100,000 overall incident rate reported by Oliver at al., for the period July 2016 to June 2018 inclusive(1)

Additional Table 4: 2019 national extrapolation of iGAS disease incidence and healthcare costs by age group for Aboriginal and Torres Strait Islander children in Australia

|  | **<1 year** | **1-4 years** | **5-9 years** | **10-14 years** | **15-17 years** | **Combined <18 years^1^**  **Rate based on combined 2019 age category incidence** | **Combined <18 years^2^**  **Rate based on historic overall incidence** |
| --- | --- | --- | --- | --- | --- | --- | --- |
| Population (2019 - modelled)^3^ | 20,048 | 79,599 | 99,677 | 92,017 | 53,442 | 344,784 | 344,784 |
| Incident rate (per 100,000) | 19.13 | 8.39 | 1.21 | 0.41 | 0 | 3.51 | 3.43 |
| Impacted population | 3.8 | 6.7 | 1.2 | 0.4 | 0.0 | 12.1 | 11.8 |
| **Prior to index admission** | | | | | | | |
| Prior ED consultations | $2,706.10 | $4,712.25 | $851.02 | $266.20 | $0.00 | $8,535.57 | $8,344.49 |
| Prior GP consultations | $488.41 | $850.49 | $153.60 | $48.05 | $0.00 | $1,540.54 | $1,506.05 |
| **During index admission** | | | | | | | |
| Total index admission | $230,540.98 | $401,450.21 | $72,500.74 | $22,678.49 | $0.00 | $727,170.42 | $710,891.53 |
| **Inpatient re-admissions (6 months post index admission)** | | | | | | | |
| Rehabilitation admissions | $13,905.95 | $24,214.98 | $4,373.16 | $1,367.94 | $0.00 | $43,862.03 | $42,880.11 |
| Re-admissions | $11,623.82 | $20,241.03 | $3,655.47 | $1,143.44 | $0.00 | $36,663.77 | $35,842.99 |
| **Outpatient services (6 months post index admission)** | | | | | | | |
| Outpatient consultations | $756.03 | $1,316.50 | $237.76 | $74.37 | $0.00 | $2,384.66 | $2,331.28 |
| **TOTAL HEALTH CARE COSTS PRE, INDEX ADMISSION AND THE 6 MONTHS FOLLOWING** | | | | | | | |
| Total health care costs | $260,021.30 | $452,785.46 | $81,771.74 | $25,578.49 | $0.00 | $820,156.99 | $801,796.45 |

^1^ Combining the individual age group categories to create an overall population and incident rate

^2^ Using the 3.43/100,000 (corrected) overall incidence rate reported by Oliver et al., for the period 1 July 2016 to 30 June 2018 inclusive (1)

^3^ Individual year-based population numbers were only available for 2016. However, the overall population grew from 798,365 in 2016 to 847,992 in 2019 (6.2% growth). Therefore, this population growth rate was applied to the 2016 data to obtain individual year-based populations number for 2019.
